# Multimodal Imaging for Cervical Aggressive Fibromatosis: Diagnosis and Individualized Interventional Therapy

**DOI:** 10.1002/ccr3.73789

**Published:** 2026-11-10

**Authors:** Ying Wang, Yan Hu, Meimei Han, Shuang Yang

**Affiliations:** ^1^ Graduate School Tianjin Medical University Tianjin China; ^2^ Department of Ultrasound Zhongshan Hospital Affiliated to Dalian University Dalian Liaoning China; ^3^ Department of Pathology Zhongshan Hospital Affiliated to Dalian University Dalian Liaoning China

**Keywords:** aggressive fibromatosis, cervical desmoid tumor, contrast‐enhanced ultrasound, CT‐guided radioactive seed implantation, multimodal imaging, positron emission tomography‐computed tomography

## Abstract

Multimodal imaging, including contrast‐enhanced ultrasound, is crucial for characterizing cervical aggressive fibromatosis. When surgery poses a high risk, imaging findings can support alternative strategies like CT‐guided brachytherapy, offering a safe and effective treatment option for this locally invasive tumor.

## 1 Introduction

Aggressive fibromatosis (AF), also known as desmoid tumors, is a rare, locally invasive soft tissue neoplasm arising from musculoaponeurotic structures. Its annual incidence is estimated at 2–4 cases per million [1, 2]. AF can occur anywhere in the body and is typically classified as extra‐abdominal, intra‐abdominal, or abdominal wall AF. Head and neck AF is relatively uncommon, comprising less than 6%–8% of all AF cases and about 7%–25% of all extra‐abdominal cases [3]. Notably, the risk of recurrence for cervical AF is higher than for non‐head and neck lesions [4]. The nonspecific clinical presentation, frequently a painless or painful mass, coupled with the lesion's rarity, often leads to diagnostic delay. Moreover, the intricate cervical anatomy and the tumor's infiltrative growth pattern render complete surgical resection without compromising vital neurovascular structures exceedingly difficult, complicating treatment decision‐making [3]. Herein, we present a case of cervical AF, detailing its multimodal imaging features and discuss the successful application of CT‐guided brachytherapy, thereby underscoring the pivotal role of imaging in individualizing treatment for this challenging condition.

## 2 Case Presentation

A 68‐year‐old postmenopausal female presented with a recently enlarged and mildly tender left cervical mass, initially discovered incidentally. There was no redness or swelling of the overlying skin, and the patient experienced discomfort and pain with movement of the mass, and the mass showed a gradual increase in recent months. Her menarche occurred at age 14. She is postmenopausal and has a history of childbearing. She had no known gynecologic pathology. She had a long‐term history of hypertension, which was well controlled with oral antihypertensive drugs, and a history of renal insufficiency requiring hemodialysis three times weekly.

### 2.1 Physical Examination

A left cervical mass measuring approximately 5 × 5 × 5 cm was palpable, with hard consistency, poor mobility, and mild tenderness on palpation; no obvious enlargement of superficial lymph nodes was found in the neck.

### 2.2 Laboratory Examinations

Serum tumor markers including carcinoembryonic antigen, alpha‐fetoprotein, carbohydrate antigen 19–9, carbohydrate antigen 125, and squamous cell carcinoma‐related antigen were all within the normal reference range.

### 2.3 Imaging Examinations

1. Ultrasound examination: Two‐dimensional ultrasound showed a hypoechoic mass in the left cervical region, with a clear boundary, irregular contour, and heterogeneous internal echotexture. Color Doppler flow imaging showed visible blood flow signals inside and around the mass (Figure 1). Contrast‐enhanced ultrasound (CEUS) showed that the contrast agent presented heterogeneous hyperenhancement from the periphery toward the center (Figure 2). Ultrasound findings suggested a solid space‐occupying lesion in the left cervical region; malignancy could not be ruled out.
2. MRI examination: 3.0 T non‐contrast cervical MRI showed a mass adjacent to the left trapezius muscle and splenius capitis muscle at the C4–C7 level, with isointense signal on T1‐weighted imaging (T1WI), slightly hyperintense signal on T2‐weighted imaging (T2WI), and hyperintense signal on T2‐fat suppression imaging; the internal signal of the mass was heterogeneous, with visible traversing vascular signal voids. Enhanced MRI was recommended for further evaluation (Figure 3).
3. PET/CT examination: A lobulated soft tissue density shadow was seen in the left posterior neck (at the C5–T1 level), with indistinct borders and poor demarcation from the adjacent left erector spinae and trapezius muscles. The largest cross‐sectional dimension was approximately 4.9 × 4.5 × 5.0 cm, showing focally increased radioactive tracer uptake with a SUVmax of 3.9 (Figure 4). The findings suggested a soft tissue mass in the left posterior neck, invading the left erector spinae and trapezius muscles, with increased metabolism: consistent with aggressive fibromatosis.

**FIGURE 1:**
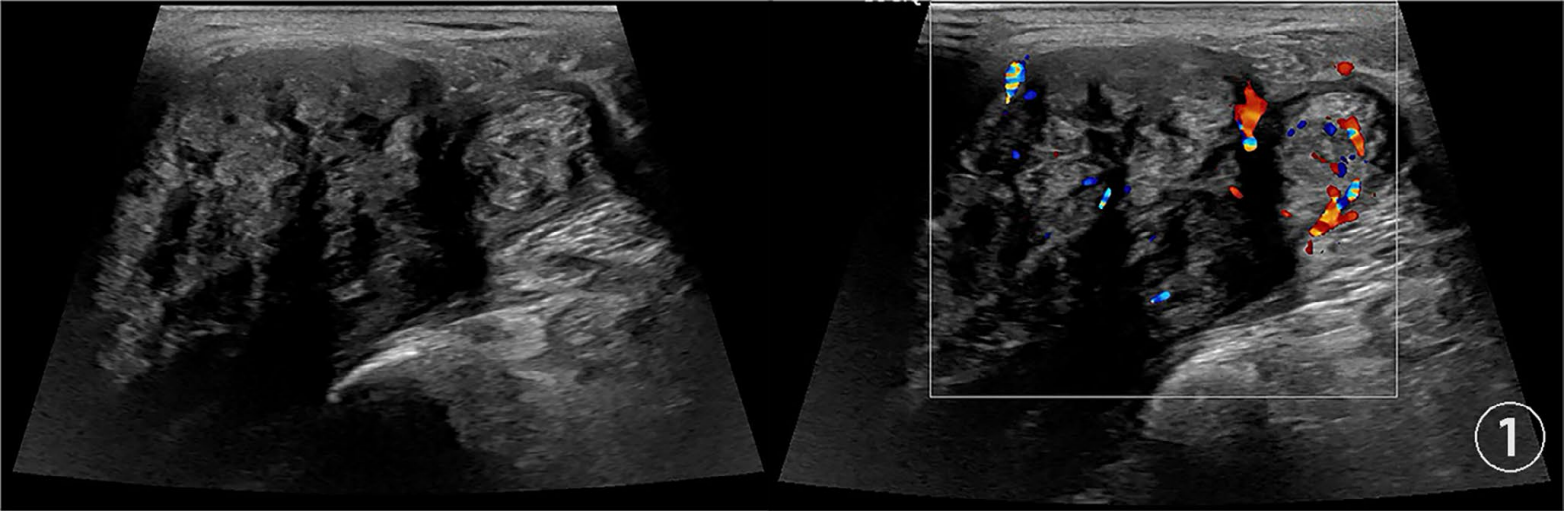
Left cervical lesion on two‐dimensional and color Doppler ultrasound. The mass appears hypoechoic with irregular contour, and blood flow signals are detectable in and around the lesion.

**FIGURE 2:**
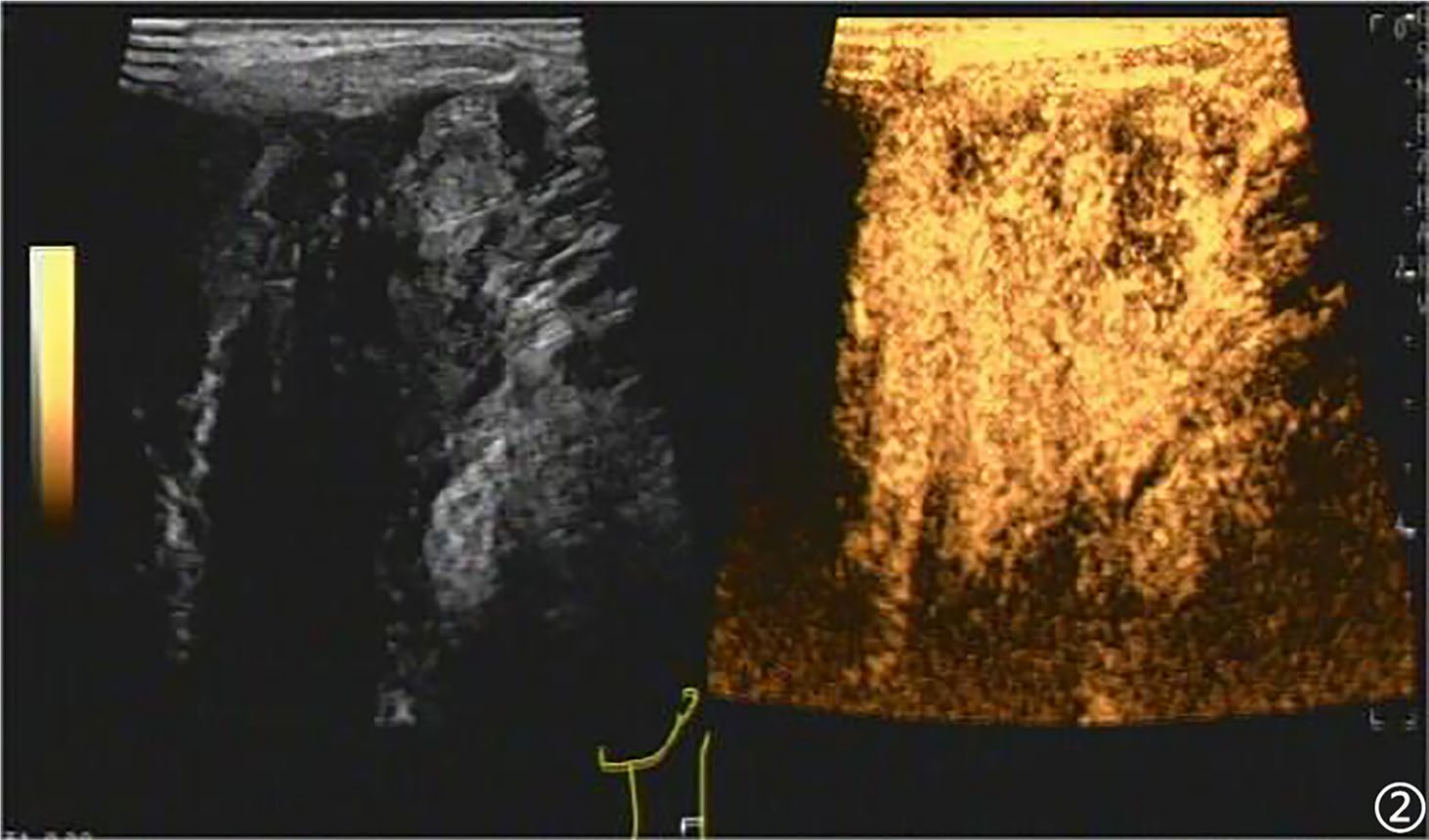
CEUS of the left cervical lesion. The lesion exhibits heterogeneous hyperenhancement from the periphery to the center with contrast agent perfusion.

**FIGURE 3:**
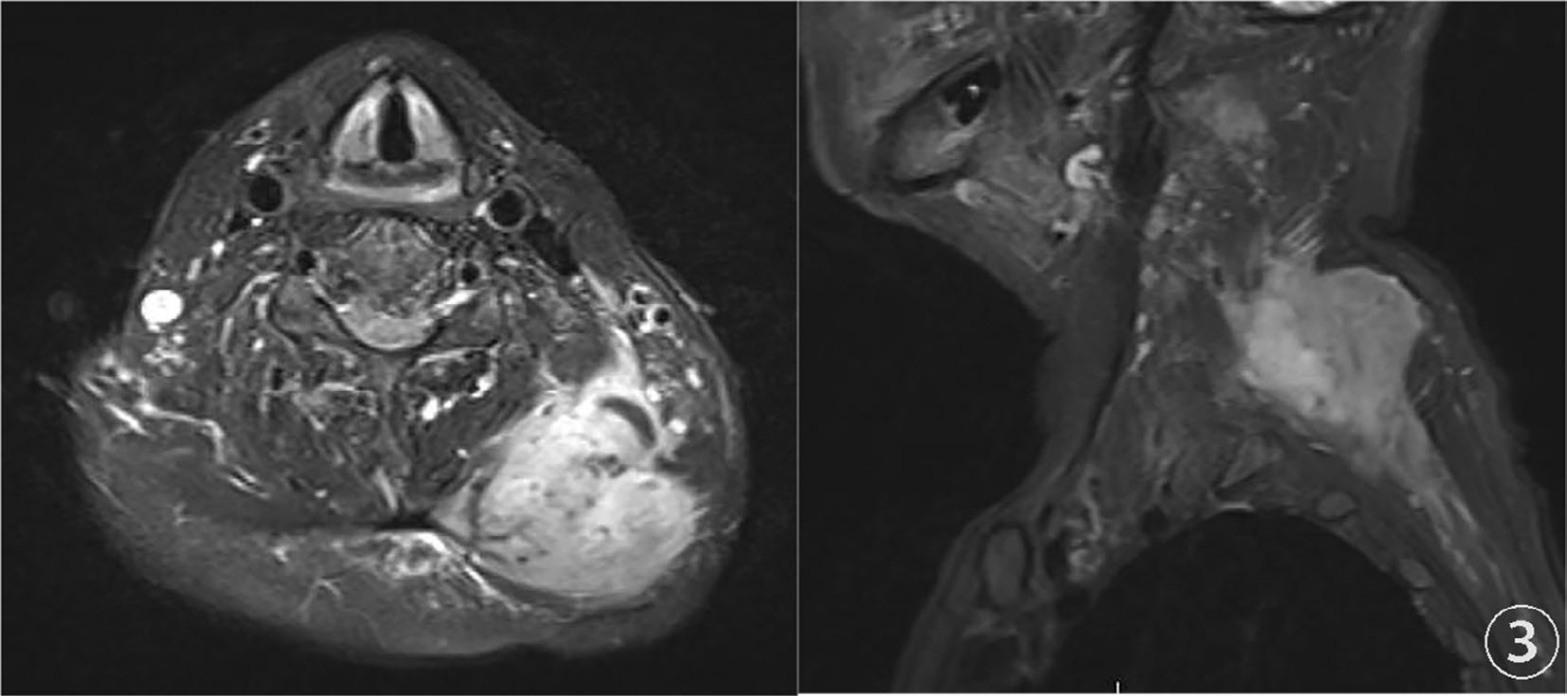
C4–C7 cervical spine T2‐weighted fat‐suppressed MRI. A space‐occupying lesion adjacent to the left trapezius and splenius capitis muscles shows slight hyperintensity and heterogeneous internal signal.

**FIGURE 4:**
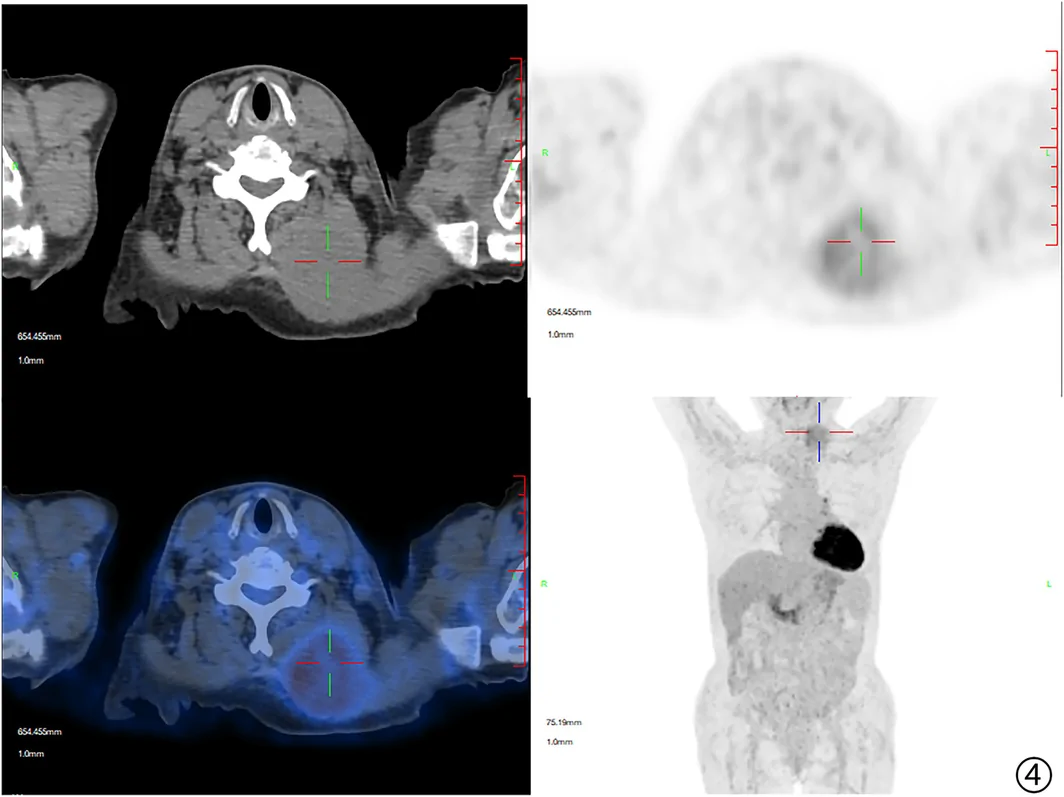
C5–T1 left posterior cervical PET/CT fusion image. A lobulated soft tissue mass with ill‐defined borders has focal increased tracer uptake, with SUVmax measured at 3.9.

### 2.4 Pathological Examination

Ultrasound‐guided core needle biopsy was performed on the left cervical mass, and histopathological examination showed a spindle cell tumor. Immunohistochemistry showed: CK (−), Vimentin (+), S‐100 (−), Sox10 (−), CD34 (vascular+), SMA (+), STAT6 (−), Desmin (−), β‐catenin (membranous/cytoplasmic+), CD10 (+), Calponin (+), Ki67 (1%) (Figure 5). Combined with the histopathological and immunohistochemical findings, the diagnosis of cervical aggressive fibromatosis was confirmed.

**FIGURE 5:**
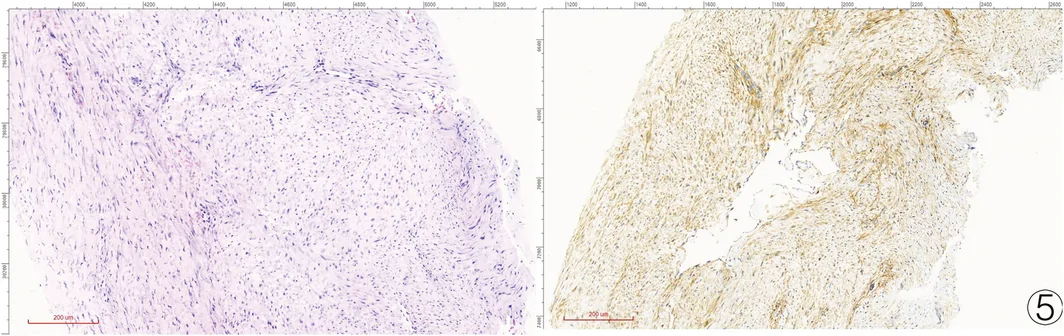
Cervical lesion histopathology and immunohistochemistry (×100). HE staining shows proliferative spindle fibroblasts with interstitial myxoid degeneration; β‐catenin (membranous/cytoplasmic) is positively expressed.

### 2.5 Treatment and Follow‐Up

Given the complex anatomical location and the high risk of postoperative nerve and vascular injury, surgical resection was deemed inappropriate following multidisciplinary discussion. The patient was treated with CT‐guided radioactive seed implantation brachytherapy, and a total of 70 radioactive seeds with an activity of 0.7 mCi each were implanted, with a total prescribed radiation dose of 110 Gy (Figure 6). The patient was followed up for 18 months after the procedure, and the cervical mass remained stable in size without obvious enlargement; no obvious adverse reactions such as radiation‐induced dermatitis and mucosal injury were found, and the patient's vital signs were stable with well‐controlled comorbidities.

**FIGURE 6:**
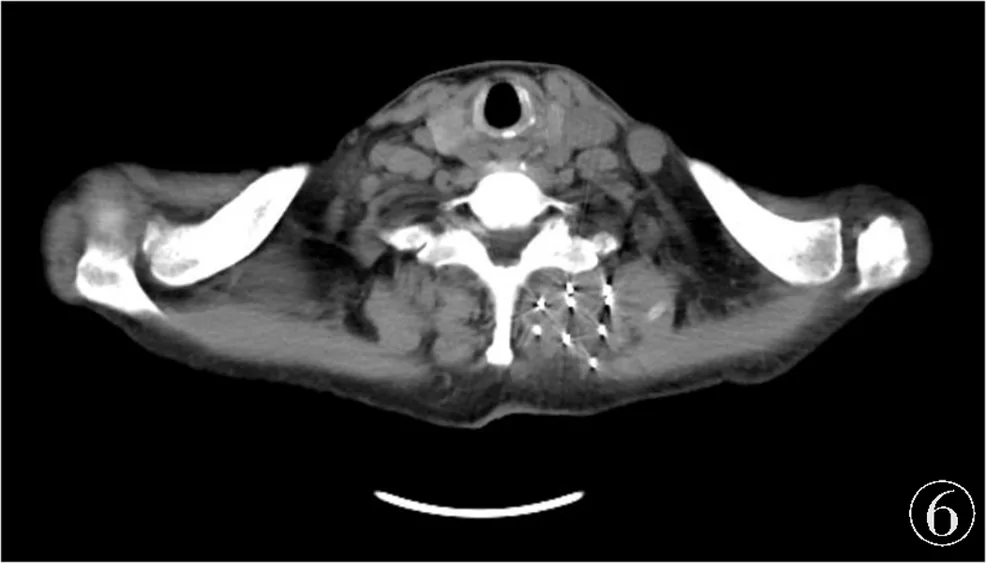
CT‐guided radioactive seed implantation for left cervical lesion. Preimplantation CT localizes the lesion; postimplantation CT reveals uniform radioactive seed distribution in the tumor target area (total prescribed dose, 110 Gy).

## 3 Discussion

Aggressive fibromatosis is a clonal fibroblastic proliferation marked by locally invasive behavior and a propensity for recurrence, yet it lacks metastatic potential [5]. Its exact etiology remains unclear, with current studies suggesting potential associations with various factors including surgery or accidental trauma, pregnancy, estrogen levels, and familial adenomatous polyposis (FAP) [6]. The disease is more common in females, can occur at any age, with peak incidence concentrated between 20–40 years and 60–69 years [7], which is consistent with the age and sex of the patient in this case.

At the molecular level, the pathogenesis of AF is closely related to aberrant activation of the Wnt/β‐catenin signaling pathway [8, 9]: familial cases often result from biallelic inactivation of the APC gene leading to impaired degradation of β‐catenin, while sporadic cases are predominantly caused by CTNNB1 gene mutations. Both lead to accumulation of β‐catenin in the cytoplasm, thereby driving tumorigenesis [10]. In this case, immunohistochemistry showed nuclear expression of β‐catenin, consistent with literature reports [11, 12]. Furthermore, AF typically shows positive immunohistochemical staining for SMA and Vimentin and is negative for Desmin, S‐100, and often other markers like the one tested here [13]; the pathological results of this case were in line with this feature, which further supported the diagnosis.

The nonspecific clinical manifestation of cervical AF makes clinical diagnosis difficult [3], and multimodal imaging plays an irreplaceable role in its early diagnosis, lesion characterization, and preoperative evaluation [14, 15].

Ultrasound is the first choice for the initial screening of cervical masses due to its noninvasiveness, real‐time capability and repeatability; two‐dimensional ultrasound can show the size, boundary, internal echo and adjacent tissue relationship of the tumor, and color Doppler can evaluate the blood supply of the lesion, while CEUS can further reflect the perfusion characteristics of the tumor microvasculature, which is helpful for distinguishing benign and malignant lesions [16, 17]. In this case, the AF lesion showed heterogeneous hypoechoic and peripheral‐to‐central heterogeneous hyperenhancement on CEUS, which was related to the composition of collagen fiber, fibrous tissue and tumor cells in the lesion [18, 19].

On CT imaging, cervical AF typically presents as a soft tissue mass with ill‐defined borders, whose density is slightly higher than that of skeletal muscle with heterogeneous internal density reflecting collagen and myxoid components [14, 15]. Post‐contrast mild to moderate enhancement is a typical feature with rare necrotic areas [20], and the “artery penetration sign” on contrast‐enhanced CT is a key feature to distinguish AF from cervical malignant soft tissue tumors [21].

MRI has high soft‐tissue resolution and is the gold standard for evaluating the extent of AF invasion and the relationship with adjacent structures; the signal characteristics of AF on MRI are closely related to its tissue composition, with fibrous and collagen‐rich areas showing low signal on T2WI and mild to moderate enhancement in the delayed phase, and cell and myxoid component‐rich areas showing high signal on T2WI and moderate to marked enhancement in the arterial phase [22, 23]. The “band‐like sign” on MRI is a relatively characteristic manifestation of AF, with an incidence of 60%–90%, which has certain diagnostic value [3]. PET/CT can evaluate the metabolic activity of the tumor and the presence of multifocal lesions [21], and the mild to moderate increase of SUVmax in AF can be used to distinguish it from malignant soft tissue tumors with high metabolic activity [14, 24]. The combination of multiple imaging modalities can make up for the deficiencies of a single examination and enable comprehensive evaluation of cervical AF.

In recent years, the treatment strategy for AF has shifted from a surgery‐first approach to individualized management centered on active surveillance (AS) [25, 26]. Current guidelines recommend AS as the preferred initial strategy for asymptomatic or minimally symptomatic AF patients [1, 27, 28]. Multiple international multicenter prospective studies have demonstrated that approximately two‐thirds of AF patients can be effectively managed with AS alone over a 5‐year follow‐up period, with some tumors even undergoing spontaneous regression [25, 29, 30]; however, approximately 30% of patients ultimately require active treatment due to tumor progression [29, 31]. In our case, the AF was located in the neck, adjacent to the trachea and major blood vessels, with limited cervical space such that even minor progression could compromise the airway and threaten life. Moreover, the patient presented with progressive tumor enlargement accompanied by pain and restricted mobility prior to presentation, and had concomitant renal insufficiency requiring thrice‐weekly hemodialysis, rendering her a poor surgical candidate per multidisciplinary evaluation. Therefore, CT‐guided radioactive seed implantation brachytherapy was selected as the initial treatment, which is clinically well justified. Literature suggests that radiotherapy alone can provide adequate local control in most patients with progressive disease who are not surgical candidates and whose disease is refractory to medical therapy [27].

In addition to local therapies, systemic therapy is also an important component of AF management [26]. With advancing understanding of the molecular biology of AF and the application of tyrosine kinase inhibitors (TKIs) in AF treatment, cytotoxic chemotherapy has gradually been relegated to a secondary role [32]. Current systemic treatment options, including TKIs, γ‐secretase inhibitors, and cytotoxic chemotherapy, have demonstrated varying degrees of antineoplastic activity: in the Phase III sorafenib trial (NCT02066181), the 24‐month progression‐free survival (PFS) rate was 81% with an objective response rate (ORR) of 33% [33]; in the DeFi Phase III trial published in 2023, nirogacestat reduced the risk of disease progression by 71% (HR 0.29) compared with placebo and significantly improved pain, physical function, and quality of life [34]. Given that our patient required chronic dialysis due to renal insufficiency, altered pharmacokinetics and additional toxicities of systemic therapy warrant particular concern. By contrast, CT‐guided radioactive seed implantation brachytherapy, as a localized treatment modality, offers precise targeting with minimal systemic toxicity [35], making it more suitable for this patient's individualized treatment needs. At 18 months post‐procedure, the cervical mass remained stable in size without significant enlargement, and no notable adverse reactions such as radiation dermatitis or mucosal injury were observed, suggesting that this treatment approach is safe and feasible in this high‐risk patient.

## 4 Conclusion

This study reports a rare case of cervical aggressive fibromatosis in a 68‐year‐old female patient and clarifies the characteristic imaging manifestations of the lesion on ultrasound, MRI, and PET/CT. Multimodal imaging can accurately evaluate the pathological features and invasive range of cervical AF, which is the basis for formulating individualized treatment plans. For cervical AF patients with high surgical risk, CT‐guided radioactive seed implantation is a safe and effective minimally invasive interventional treatment method, which can achieve good local tumor control and provide a new clinical option for the treatment of this rare disease. Larger sample size and longer follow‐up studies are needed to further verify the long‐term efficacy of radioactive seed implantation in the treatment of cervical AF.

## Author Contributions

**Ying Wang:** writing – original draft, conceptualization. **Yan Hu:** writing – review and editing. **Meimei Han:** investigation, data curation. **Shuang Yang:** writing – review and editing, funding acquisition, resources.

## Funding

This work was supported by the Dalian Municipal Guided Program in the Field of Life and Health, China, 2024 (2024ZDJH01PT153).

## Ethics Statement

This case report was approved by the Research Ethics Committee of The Affiliated Zhongshan Hospital of Dalian University.

## Consent

Written informed consent was obtained from the patient for publication of this case report and any accompanying images. A copy of the consent is available for review by the Editor‐in‐Chief of this journal upon request.

## Conflicts of Interest

The authors declare no conflicts of interest.

## Data Availability

The data that support the findings of this case report are available from the corresponding author upon reasonable request, subject to privacy and ethical restrictions.
